# Association between chronic kidney disease and age-related macular degeneration: a Mendelian randomization study

**DOI:** 10.3389/fnagi.2024.1399666

**Published:** 2024-05-30

**Authors:** Yawei Hou, Qinglin Liu, Zhenwei Xiao, Yameng Li, Xinyang Tian, Zhenguo Wang

**Affiliations:** ^1^Institute of Chinese Medical Literature and Culture, Shandong University of Traditional Chinese Medicine, Jinan, China; ^2^College of Traditional Chinese Medicine, Shandong University of Traditional Chinese Medicine, Jinan, China; ^3^Department of Nephrology, Affiliated Hospital of Shandong University of Traditional Chinese Medicine, Jinan, China; ^4^The First Clinical Medical College, Shandong University of Traditional Chinese Medicine, Jinan, China

**Keywords:** chronic kidney disease, age-related macular degeneration, diabetic nephropathy, IgA nephropathy, membranous nephropathy, estimated glomerular filtration rate, Mendelian randomization

## Abstract

**Purpose:**

Observational studies have reported inconsistent results on the relationship between chronic kidney disease (CKD) and age-related macular degeneration (AMD). The primary objective of this study was to investigate the causal relationships between estimated glomerular filtration rate (eGFR), CKD, its common causes, and AMD among participants of European descent.

**Methods:**

Genetic variants associated with eGFR, CKD and its common causes, encompassing diabetic nephropathy (DN), immunoglobulin A nephropathy (IgAN), and membranous nephropathy (MN) were obtained from previously published genome-wide association studies (GWAS) and FinnGen database. Summary statistics for early AMD, AMD, dry AMD, and wet AMD were acquired from the GWAS and FinnGen database. Inverse-variance-weighted (IVW) method was the main MR analysis. Sensitivity analyses were performed with Cochran’s Q, MR-Egger intercept, and leave-one-out analysis. In addition, RadialMR was utilized to identify and remove outliers.

**Results:**

IVW results showed that CKD, eGFR were not associated with any type of AMD (*p* > 0.05). DN (OR: 1.042, 95% CI: 1.002–1.083, *p* = 0.037) and MN (OR: 1.023, 95% CI: 1.007–1.040, *p* = 0.005) were associated with an increased risk of earl AMD. DN (OR: 1.111, 95% CI: 1.07–1.154, *p* = 4.87 × 10^−8^), IgAN (OR: 1.373, 95% CI: 1.097–1.719, *p* = 0.006), and MN (OR: 1.036, 95% CI: 1.008–1.064, *p* = 0.012) were associated with an increased risk of AMD. DN (OR: 1.090, 95% CI: 1.042–1.140, *p* = 1.57 × 10^−4^) and IgAN (OR: 1.480, 95% CI: 1.178–1.858, *p* = 7.55 × 10^−4^) were associated with an increased risk of dry AMD. The risk of wet AMD was associated with DN (OR: 1.107, 95% CI: 1.043–1.174, *p* = 7.56 × 10^−4^) and MN (OR: 1.071, 95% CI: 1.040–1.103, *p* = 5.48 × 10^−6^).

**Conclusion:**

This MR study found no evidence of causal relationship between CKD and AMD. DN, IgAN, and MN may increase risk of AMD. This findings underscore the importance of ocular examinations in patients with DN, MN, and IgAN. More studies are needed to support the findings of our current study.

## 1 Introduction

Age-related macular degeneration (AMD) stands as the principal cause of non-reversible visual impairment among the elderly, with projections suggesting an affected population of approximately 288 million globally by 2040 (Wong W. L. et al., 2014). The condition not only imposes a substantial economic impact but also significantly diminishes the quality of life (Jonas, 2014). AMD is clinically stratified into early, intermediate, or advanced stages, and further distinguished by the presence of neovascularization as either wet (neovascular) or dry (non-neovascular) AMD. The latter, dry AMD, represents the more prevalent form and may evolve into the more detrimental wet AMD, which is responsible for roughly 80% of severe vision loss from AMD due to retinal hemorrhage and exudation (Lim et al., 2012; Gil-Martínez et al., 2020). Despite considerable progress in comprehending AMD, the precise causes remain partially elusive. Consequently, early detection and timely intervention are crucial for preserving functional vision.

Chronic kidney disease (CKD) represents a condition of growing prevalence, which escalates notably with advancing age. As an emergent public health concern, CKD affects an estimated 9–16% of the global population (Coresh et al., 2003). Notably, there is evidence to suggest that CKD and AMD may share overlapping risk factors (Haroun et al., 2003; Heesterbeek et al., 2020) and pathophysiological pathway (Mullins et al., 2001; Haines et al., 2005; Xing et al., 2008). Recent research has indicated a potential link between these two diseases. Nonetheless, the literature presents divergent results; while some studies have identified an increased risk of AMD in patients with CKD (Liew et al., 2008; Weiner et al., 2011; Cheung et al., 2014), others have not established a connection (Park et al., 2014; Wong et al., 2016; Zhu et al., 2020). Consequently, the existence of a definitive association between CKD and AMD is still unresolved.

Mendelian randomization (MR) analysis leverages genetic variation as an instrumental variables (IVs) to assess the potential causal links between risk factors and diseases (Lawlor et al., 2008; Burgess et al., 2017). This method capitalizes on the principle that genetic variants are assigned randomly at conception and remain uninfluenced by typical confounders. Due to this random allocation, MR analysis is less prone to biases from confounders and reverse causation than are traditional observational studies. In this study, we employed a two-sample MR approach to explore the causal relationships between renal function, CKD, its common etiologies [diabetic nephropathy (DN), IgA nephropathy (IgAN), membranous nephropathy (MN)], and diverse forms of AMD including early AMD, overall AMD, dry AMD, and wet AMD, specifically in a European population.

## 2 Methods

### 2.1 Study design

**Figure 1:**
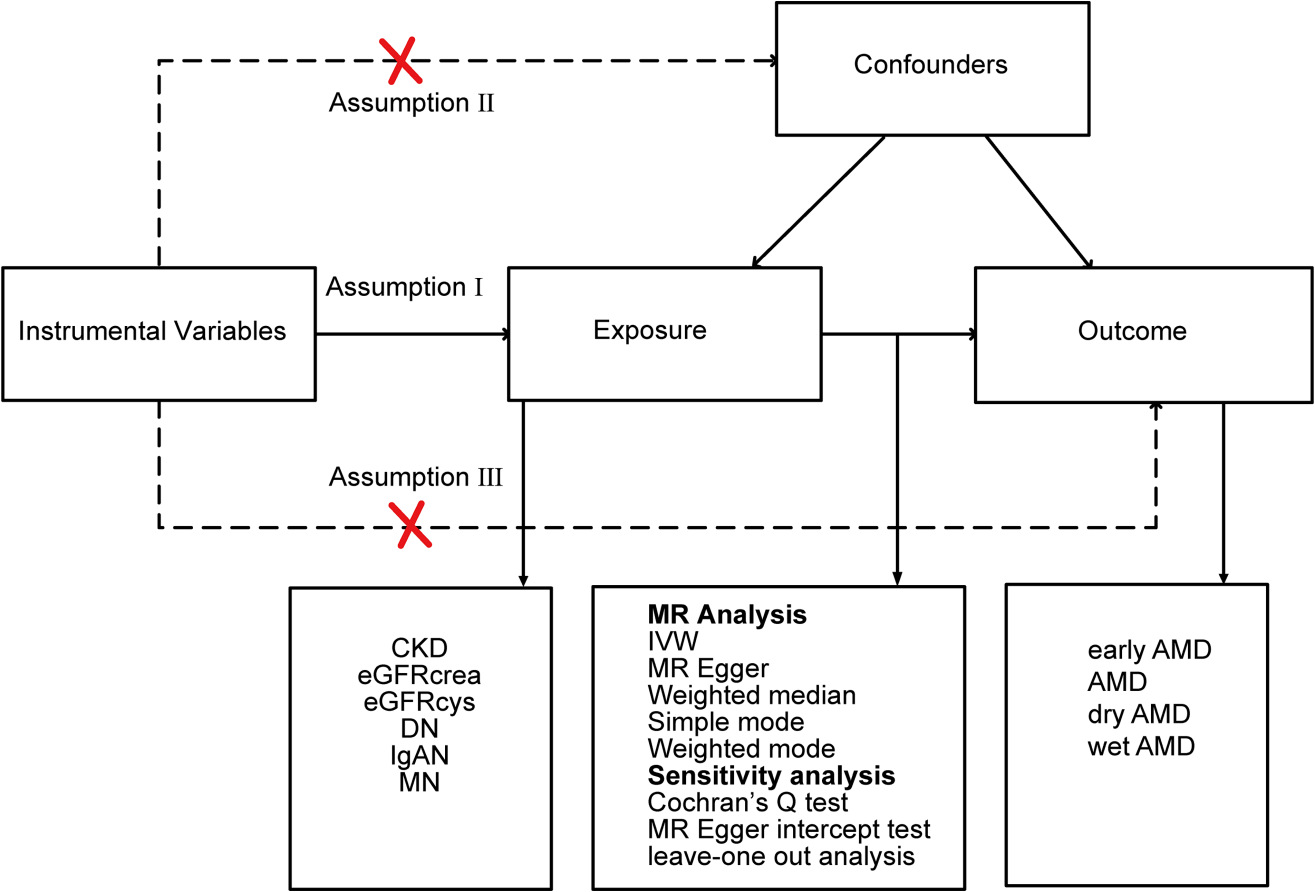
Study design overview. CKD, chronic kidney disease; eGFRcrea, creatinine-based estimated glomerular filtration rate; eGFRcys, cystatinC-based estimated glomerular filtration rate; DN, diabetic nephropathy; IgAN, immunoglobulin A nephropathy; MN, membranous nephropathy; IVW, inverse-variance weighted; AMD, age-related macular degeneration.

We employed a two-sample MR, leveraging summary-level genetic associations from diverse genome-wide association studies (GWAS). A robust MR framework adheres to three critical assumptions: (1) IVs exhibit strong associations with the exposures; (2) IVs are not linked with any confounders; (3) IVs affect the outcome solely via the exposures under consideration. Considering the chronic progression and complex etiology of CKD, our analysis encompassed multiple phenotypes. These encompassed CKD (defined as an estimated glomerular filtration rate (eGFR) below 60 mL/min/1.73 m^2^), creatinine-based eGFR (eGFRcrea), cystatin C-based eGFR (eGFRcys), and particular renal diseases, namely DN, IgAN, and MN, which are prevalent causes of CKD. Regarding AMD, we examined four subtypes: early AMD, AMD (whether dry or wet), dry AMD (inclusive of geographic atrophy), and wet AMD. The study design overview is presented in Figure 1.

### 2.2 Data source of exposure

The GWAS summary statistics for CKD were obtained from a meta-analysis by the CKDGen Consortium, which included data from 23 cohorts of European descent (comprising 480,698 individuals; among them, 41,395 were CKD patients and 439,303 were controls) (Wuttke et al., 2019). The summary statistics for eGFRcrea and eGFRcys were derived from a separate meta-analysis (Stanzick et al., 2021) that combined information from the CKDGen Consortium and the UK Biobank. For DN, summary-level GWAS data were sourced from the FinnGen database, which featured 312,650 participants of European ancestry, including 4,111 DN cases and 308,539 controls (Kurki et al., 2023). The GWAS meta-analysis for IgAN integrated data from FinnGen, the UK Biobank, and the Biobank Japan, accounting for 477,784 individuals of European background (with 15,587 cases and 462,197 controls) and 175,359 individuals of East Asian descent (with 71 cases and 175,288 controls) (Sakaue et al., 2021). Our analysis focused on the GWAS data from the European cohorts. For MN, we utilized the most recent GWAS data, which included 2,150 MN cases and 5,829 controls, all of European ancestry (Xie et al., 2020). These participants were all diagnosed with primary MN. Furthermore, the cohort underwent genotyping with high-density SNP arrays, and approximately 7 million common, high-quality genetic markers were imputed using the most up-to-date genome-wide sequence reference panel. Supplementary Table S1 presents additional details regarding each dataset.

### 2.3 Data source of outcome

The GWAS summary data for early AMD were obtained from a recently published genome-wide association meta-analysis, encompassing 14,034 cases and 91,214 controls, all of whom are of European descent (Winkler et al., 2020). The summary-level data for AMD, including AMD (whether dry or wet), dry AMD (includes geographic atrophy), and wet AMD, were derived from the tenth round of analysis from the FinnGen (Kurki et al., 2023). Supplementary Table S1 provides further details on each dataset.

### 2.4 Selection of instrumental variables (IVs)

To adhere to the three fundamental assumptions of MR and guarantee the precision of our findings, we employed single nucleotide polymorphisms (SNPs) as IVs after a thorough quality control process. First, we identified SNPs that were strongly associated with the exposures in each MR analysis, indicated by a *p*-value threshold of less than 5 × 10^−8^. Second, we subsequently carried out a clumping procedure to select independent SNPs using a linkage disequilibrium threshold (*r*^2^ < 0.001, clumping distance = 10,000 kb). For MN, a more lenient threshold was used (*r*^2^ < 0.01, clumping distance = 1,000 kb) to ensure a sufficient number of SNPs. SNPs associated with the outcomes at a significance level of *p* < 5 × 10^−8^ were excluded. Third, we utilized the Phenoscanner database (Staley et al., 2016) to scrutinize potential confounding factors linked to the SNPs, excluding any that were associated with known confounders such as smoking, alcohol consumption, hypertension, and obesity. Then, we excluded SNP with *F* values <10 to ensure the strength of the association between SNP and exposure factors (Burgess and Thompson, 2011). The F statistic is computed using the formula F = R^2^(n−k−1)/[k(1−R^2^)]. In order to prevent the influence of alleles on results for the causal relationship between CKD and AMD, palindrome SNPs were removed. We also applied Steiger filtering to discard SNPs that exhibited a stronger correlation with the outcomes than with the exposures (Hemani et al., 2017). Finally, we eliminated outlier SNPs as identified by Radial MR analysis, further refining our selection of IVs (Bowden et al., 2018).

### 2.5 MR analysis

To investigate the potential causal link between the exposure and the outcome, we applied a suite of MR methods, including inverse variance weighted (IVW), MR-Egger, weighted median, simple mode, and weighted mode approaches. The IVW method, chosen as the primary method for our analysis, presupposes the absence of horizontal pleiotropy, under the assumption that either all the SNPs being utilized are valid instrumental variables or that any pleiotropic effects are counterbalanced (Burgess et al., 2013). The weighted median method provides a reliable estimate if a majority (at least 50%) of the IVs are valid (Bowden et al., 2016). Weighted mode is less capable of detecting causal effects, but also have fewer biases (Hartwig et al., 2017). Even if most IVs have pleiotropy, MR Egger can provide effective estimates (Bowden et al., 2015). We assessed heterogeneity using the Cochran Q statistic (Greco et al., 2015), and examined the potential for horizontal pleiotropy using the MR-Egger intercept (Bowden et al., 2015). We also performed the leave-one-out analysis by eliminating SNPs one by one and recomputing the effect. All statistical analyses were conducted using R version 4.2.3 and the “TwoSampleMR,” and “RadialMR” packages. A two-sided *p* value of <0.05 was considered significant.

## 3 Results

### 3.1 The causal relationship between CKD and early AMD

**Figure 2:**
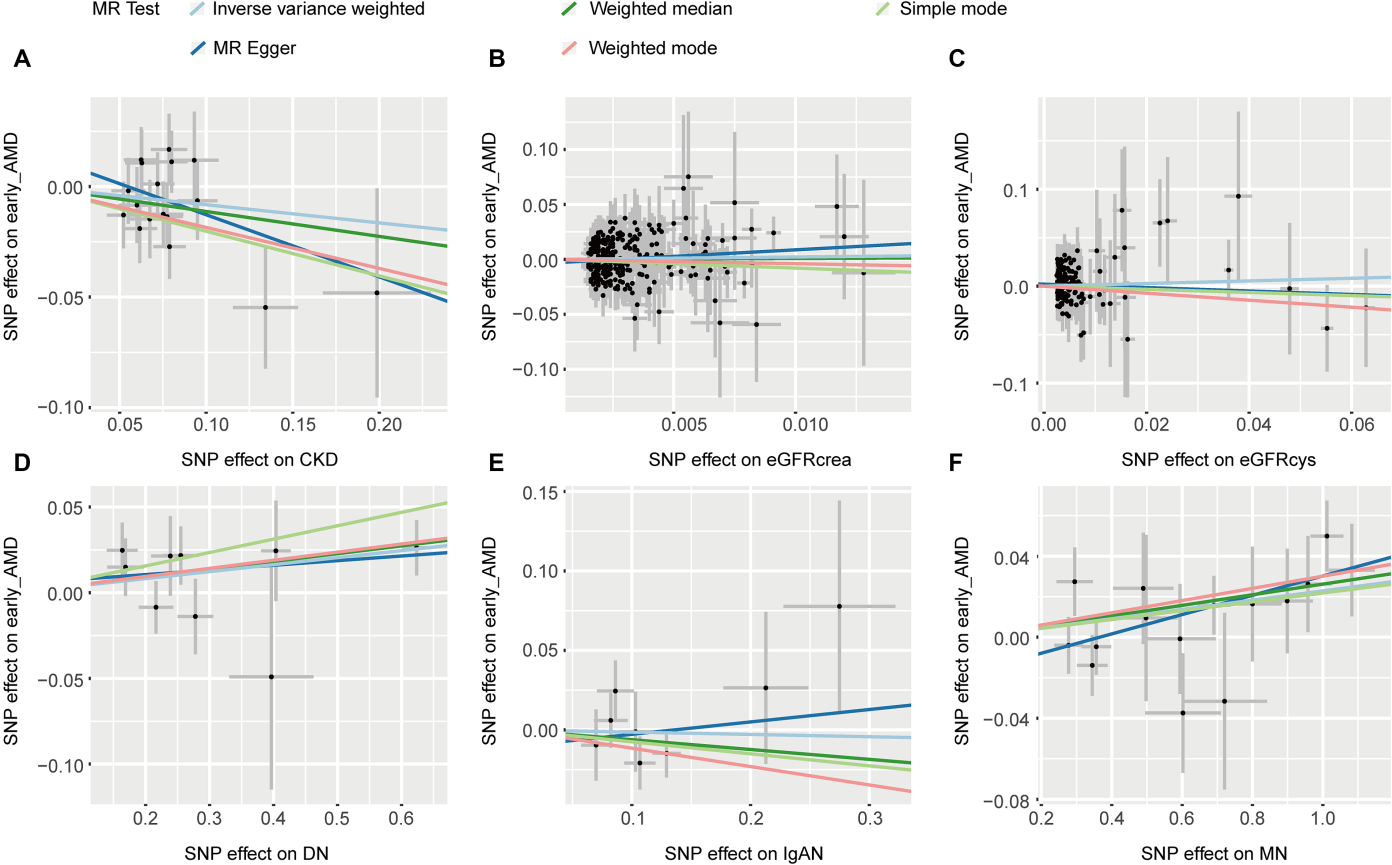
The scatter plots of CKD with the risk of early AMD. **(A)** The MR estimate for the effect of CKD on the risk of early AMD; **(B)** The MR estimate for the effect of eGFRcrea on the risk of early AMD; **(C)** The MR estimate for the effect of eGFRcys on the risk of early AMD; **(D)** The MR estimate for the effect of DN on the risk of early AMD; **(E)** The MR estimate for the effect of IgAN on the risk of early AMD; **(F)**. The MR estimate for the effect of MN on the risk of early AMD. CKD, chronic kidney disease; eGFRcrea, creatinine-based estimated glomerular filtration rate; eGFRcys, cystatinC-based estimated glomerular filtration rate; DN, diabetic nephropathy; immunoglobulin A nephropathy, IgAN; MN, membranous nephropathy; AMD, age-related macular degeneration; MR, Mendelian randomization; SNP, single nucleotide polymorphism.

IVW and other MR methods demonstrated no association between CKD, eGFRcrea, eGFRcys, and IgAN with early AMD (*p* > 0.05, Table 1). However, IVW results indicated a significant increase in the risk of early AMD associated with genetically predicted DN (OR: 1.042, *p* = 0.037). Consistent effect sizes were observed across other MR methods (OR > 1, Table 1). And IVW showed a positive association between the risk of early AMD and MN (OR: 1.023, *p* = 0.005). MR-Egger, weighted median, and weighted mode analyses confirmed these findings (OR > 1, *p* < 0.05, Table 1). The scatter plots for the causal relationship between CKD and early AMD were presented in Figure 2. There was no evidence of significant heterogeneity or pleiotropy (*p* > 0.05, Supplementary Table S2). The results of leave-one-out sensitivity and single SNP risk analysis were shown in Supplementary Figures S1, S2.

**Table 1:** Associations of genetically determined CKD with early AMD and AMD.

		Outcome			
		Early AMD		AMD	
Exposure	MR method	OR (95%CI)	*P*	OR (95%CI)	*P*
CKD	MR egger	0.755 (0.490–1.164)	0.223	0.605 (0.373–0.981)	0.063
	Weighted median	0.893 (0.768–1.040)	0.145	1.054 (0.887–1.253)	0.549
	IVW	0.921 (0.830–1.022)	0.121	1.027 (0.906–1.163)	0.682
	Simple mode	0.817 (0.619–1.078)	0.172	1.097 (0.787–1.529)	0.592
	Weighted mode	0.831 (0.624–1.106)	0.223	1.038 (0.757–1.422)	0.821
eGFRcrea	MR egger	3.301 (0.766–14.235)	0.110	1.323 (0.258–6.785)	0.738
	Weighted median	1.083 (0.393–2.987)	0.878	2.999 (0.899–9.999)	0.074
	IVW	1.238 (0.648–2.367)	0.518	1.659 (0.801–3.438)	0.173
	Simple mode	0.452 (0.021–9.594)	0.611	5.522 (0.199–153.265)	0.314
	Weighted mode	0.673 (0.104–4.332)	0.677	4.829 (0.691–33.75)	0.114
eGFRcys	MR egger	0.838 (0.364–1.930)	0.679	2.039 (0.759–5.477)	0.160
	Weighted median	0.850 (0.369–1.959)	0.703	1.309 (0.519–3.301)	0.568
	IVW	1.145 (0.696–1.886)	0.594	1.004 (0.567–1.779)	0.989
	Simple mode	0.849 (0.124–5.811)	0.868	0.72 (0.093–5.584)	0.754
	Weighted mode	0.696 (0.296–1.640)	0.409	2.093 (0.672–6.516)	0.204
DN	MR egger	1.027 (0.948–1.114)	0.533	1.173 (1.078–1.276)	**0.007**
	Weighted median	1.047 (0.999–1.097)	0.056	1.120 (1.071–1.171)	**7.43E-07**
	IVW	1.042 (1.002–1.083)	**0.037**	1.111 (1.07–1.154)	**4.87E-08**
	Simple mode	1.081 (0.996–1.173)	0.098	1.108 (1.023–1.201)	**0.036**
	Weighted mode	1.048 (0.996–1.104)	0.109	1.121 (1.07–1.175)	**0.001**
IgAN	MR egger	1.081 (0.692–1.688)	0.743	1.066 (0.627–1.813)	0.829
	Weighted median	0.940 (0.793–1.114)	0.475	1.28 (0.953–1.719)	0.101
	IVW	0.986 (0.863–1.125)	0.830	1.373 (1.097–1.719)	**0.006**
	Simple mode	0.927 (0.685–1.256)	0.641	1.246 (0.832–1.866)	0.346
	Weighted mode	0.891 (0.726–1.093)	0.304	1.187 (0.787–1.79)	0.460
MN	MR egger	1.049 (1.011–1.088)	**0.024**	1.081 (1.023–1.144)	**0.023**
	Weighted median	1.027 (1.004–1.050)	**0.020**	1.050 (1.014–1.088)	**0.006**
	IVW	1.023 (1.007–1.040)	**0.005**	1.036 (1.008–1.064)	**0.012**
	Simple mode	1.022 (0.988–1.058)	0.230	1.051 (0.996–1.11)	0.102
	Weighted mode	1.031 (1.006–1.056)	**0.028**	1.052 (1.012–1.093)	**0.029**

CKD, chronic kidney disease; eGFRcrea, creatinine-based estimated glomerular filtration rate; eGFRcys, cystatinC-based estimated glomerular filtration rate; DN, diabetic nephropathy; immunoglobulin A nephropathy, IgAN; MN, membranous nephropathy; AMD, age-related macular degeneration; IVW, inverse-variance weighted.

The bold values in the tables indicate statistical significance with *P*-values less than 0.05.

### 3.2 The causal relationship between CKD and AMD

**Figure 3:**
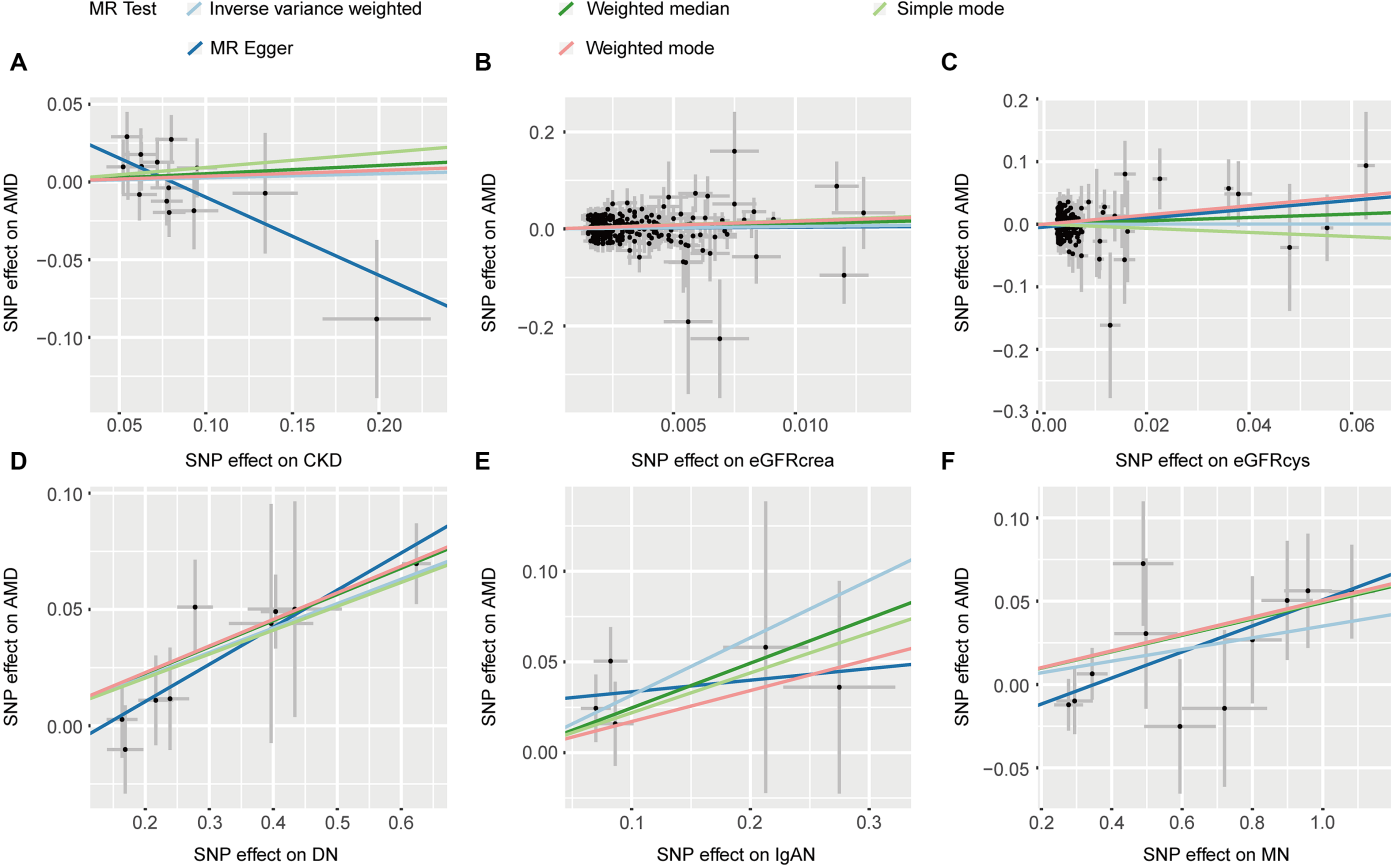
The scatter plots of CKD with the risk of AMD. **(A)** The MR estimate for the effect of CKD on the risk of AMD; **(B)** The MR estimate for the effect of eGFRcrea on the risk of AMD; **(C)** The MR estimate for the effect of eGFRcys on the risk of AMD; **(D)** The MR estimate for the effect of DN on the risk of AMD; **(E)** The MR estimate for the effect of IgAN on the risk of AMD; **(F)** The MR estimate for the effect of MN on the risk of AMD. CKD, chronic kidney disease; eGFRcrea, creatinine-based estimated glomerular filtration rate; eGFRcys, cystatinC-based estimated glomerular filtration rate; DN, diabetic nephropathy; immunoglobulin A nephropathy, IgAN; MN, membranous nephropathy; AMD, age-related macular degeneration; MR, Mendelian randomization; SNP, single nucleotide polymorphism.

Five methods consistently showed that CKD, eGFRcrea, and eGFRcys were not associated with AMD (*p* > 0.05, Table 1). IVW (OR: 1.111, *p* = 4.87 × 10^−8^) and other four MR methods consistently showed that DN induces the risk of AMD (OR > 1, Table 1). For IgAN, we found the same direction of effect size in five MR methods (OR > 1, Table 1), but only IVW method was significant (OR: 1.373, *p* = 0.006). IVW and other three methods found MN was associated with an increased risk of AMD (*p* < 0.05, Table 1). The Scatter plots of the effect of CKD on AMD were shown in Figure 3. The results from Cochrane’s Q test (Supplementary Table S2) showed that no obvious heterogeneity was found in the selected SNPs (*p* > 0.05). Furthermore, the MR-Egger tests showed that there is horizontal pleiotropy between eGFRcys and AMD (Supplementary Table S2). In the presence of pleiotropy, MR Egger should be chosen as the primary analysis method (OR: 2.039, *p* = 0.160). The results of leave-one-out sensitivity and single SNP risk analysis were shown in Supplementary Figures S3, S4.

### 3.3 The causal relationship between CKD and dry AMD

**Figure 4:**
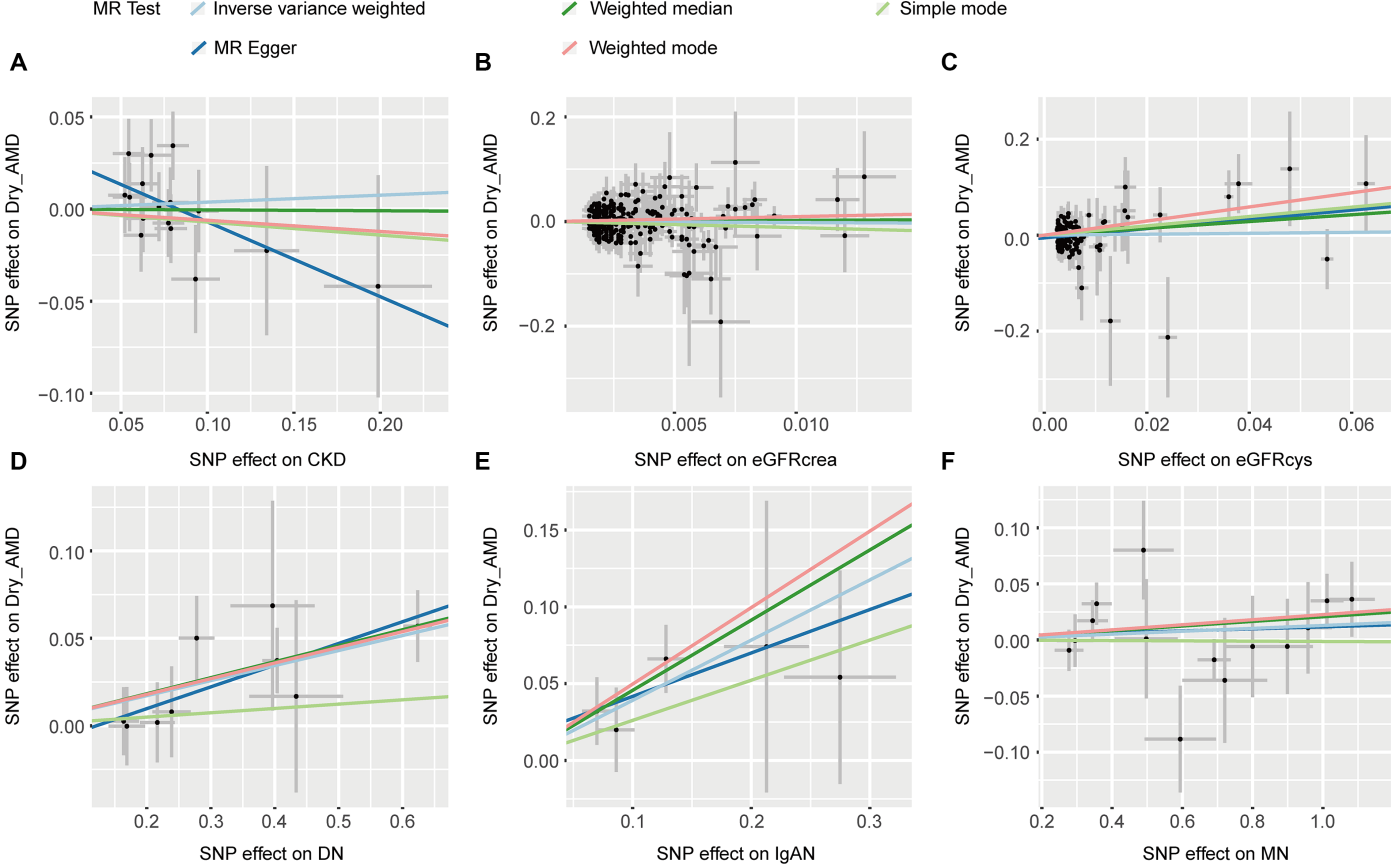
The scatter plots of CKD with the risk of dry AMD. **(A)** The MR estimate for the effect of CKD on the risk of dry AMD; **(B)** The MR estimate for the effect of eGFRcrea on the risk of dry AMD; **(C)** The MR estimate for the effect of eGFRcys on the risk of dry AMD; **(D)** The MR estimate for the effect of DN on the risk of dry AMD; **(E)** The MR estimate for the effect of IgAN on the risk of dry AMD; **(F)** The MR estimate for the effect of MN on the risk of dry AMD. CKD, chronic kidney disease; eGFRcrea, creatinine-based estimated glomerular filtration rate; eGFRcys, cystatinC-based estimated glomerular filtration rate; DN, diabetic nephropathy; immunoglobulin A nephropathy, IgAN; MN, membranous nephropathy; AMD, age-related macular degeneration; MR, Mendelian randomization; SNP, single nucleotide polymorphism.

Five MR methods, including IVW, found no association between CKD, eGFRcrea, eGFRcys, MN, and dryAMD (*p* > 0.05, Table 2). Four MR methods (IVW, MR Egger, weighted median and weighted mode) consistently showed that DN induces the risk of dry AMD (OR > 1, *p* < 0.05, Table 2). For IgAN, we found the same direction of effect size in five methods (OR > 1, Table 2). IVW (OR = 1.480, 95% CI = 1.178–1.858, *p* = 7.55 × 10^−4^; Table 2) and weighted median (OR = 1.579, 95% CI = 1.184–2.105, *p* = 1.84 × 10^−3^; Table 2) showed that IgAN induces the risk of dry AMD (Table 2). The scatter plots for the causal relationship between CKD and dry AMD were presented in Figure 4. We found no evidence of heterogeneity in our analysis (*p* > 0.05, Supplementary Table S2). MR Egger tests did not show evidence of horizontal pleiotropy (*p* > 0.05, Supplementary Table S2). The results of leave-one-out sensitivity and single SNP risk analysis were shown in Supplementary Figures S5, S6.

**Table 2:** Associations of genetically determined CKD with dry AMD and wet AMD.

		Outcome			
		Dry AMD		Wet AMD	
Exposure	MR method	OR (95%CI)	*P*	OR (95%CI)	*P*
CKD	MR egger	0.666 (0.377–1.180)	0.185	0.865 (0.399–1.874)	0.718
	Weighted median	0.995 (0.815–1.215)	0.964	1.006 (0.813–1.245)	0.958
	IVW	1.038 (0.904–1.193)	0.594	1.09 (0.934–1.273)	0.273
	Simple mode	0.932 (0.666–1.304)	0.686	0.964 (0.667–1.393)	0.846
	Weighted mode	0.941 (0.685–1.293)	0.713	0.967 (0.669–1.396)	0.859
eGFRcrea	MR egger	0.940 (0.136–6.489)	0.950	1.357 (0.146–12.599)	0.789
	Weighted median	1.235 (0.298–5.117)	0.771	1.298 (0.256–6.571)	0.753
	IVW	0.821 (0.348–1.939)	0.653	1.638 (0.620–4.326)	0.319
	Simple mode	0.310 (0.007–12.903)	0.539	1.935 (0.029–128.214)	0.758
	Weighted mode	2.547 (0.242–26.823)	0.437	1.372 (0.112–16.845)	0.805
eGFRcys	MR egger	2.622 (0.823–8.359)	0.105	2.373 (0.640–8.799)	0.198
	Weighted median	2.034 (0.693–5.973)	0.196	2.008 (0.592–6.813)	0.263
	IVW	1.103 (0.562–2.167)	0.776	0.816 (0.386–1.726)	0.595
	Simple mode	2.646 (0.161–43.526)	0.497	0.769 (0.055–10.822)	0.846
	Weighted mode	4.354 (0.889–21.335)	0.072	2.028 (0.481–8.542)	0.337
DN	MR egger	1.132 (1.025–1.251)	**0.044**	1.195 (1.053–1.355)	**0.028**
	Weighted median	1.096 (1.036–1.159)	**1.38E-03**	1.131 (1.065–1.201)	**6.59E-05**
	IVW	1.090 (1.042–1.140)	**1.57E-04**	1.107 (1.043–1.174)	**7.56E-04**
	Simple mode	1.025 (0.940–1.118)	0.592	0.983 (0.861–1.123)	0.809
	Weighted mode	1.094 (1.038–1.153)	**0.010**	1.134 (1.067–1.204)	**0.004**
IgAN	MR egger	1.326 (0.742–2.371)	0.411	1.000 (0.500–2.00)	1
	Weighted median	1.579 (1.184–2.105)	**1.84E-03**	0.948 (0.690–1.302)	0.739
	IVW	1.480 (1.178–1.858)	**7.55E-04**	0.961 (0.748–1.233)	0.752
	Simple mode	1.299 (0.878–1.920)	0.260	1.298 (0.750–2.247)	0.394
	Weighted mode	1.645 (1.144–2.364)	0.055	0.740 (0.472–1.161)	0.247
MN	MR egger	1.009 (0.954–1.067)	0.759	1.120 (1.054–1.190)	**0.004**
	Weighted median	1.021 (0.988–1.055)	0.218	1.077 (1.038–1.117)	**8.63E-05**
	IVW	1.013 (0.989–1.038)	0.298	1.071 (1.040–1.103)	**5.48E-06**
	Simple mode	0.999 (0.947–1.054)	0.961	1.094 (1.032–1.159)	**0.011**
	Weighted mode	1.023 (0.986–1.061)	0.250	1.081 (1.038–1.127)	**0.003**

CKD, chronic kidney disease; eGFRcrea, creatinine-based estimated glomerular filtration rate; eGFRcys, cystatinC-based estimated glomerular filtration rate; DN, diabetic nephropathy; immunoglobulin A nephropathy, IgAN; MN, membranous nephropathy; AMD, age-related macular degeneration; IVW, inverse-variance weighted.

The bold values in the tables indicate statistical significance with *P*-values less than 0.05.

### 3.4 The causal relationship between CKD and wet AMD

**Figure 5:**
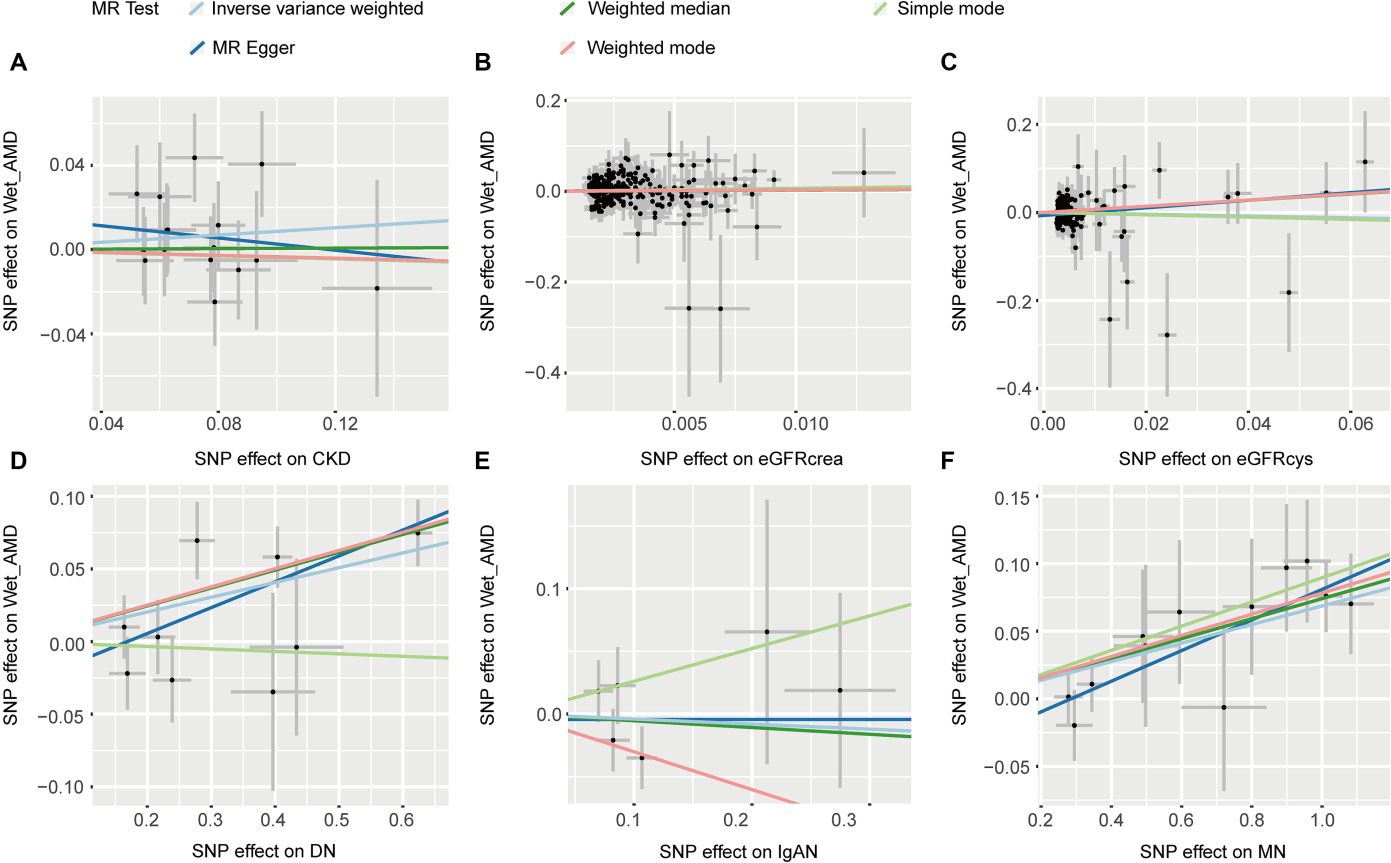
The scatter plots of CKD with the risk of wet AMD. **(A)** The MR estimate for the effect of CKD on the risk of wet AMD; **(B)** The MR estimate for the effect of eGFRcrea on the risk of wet AMD; **(C)** The MR estimate for the effect of eGFRcys on the risk of wet AMD; **(D)** The MR estimate for the effect of DN on the risk of wet AMD; **(E)**. The MR estimate for the effect of IgAN on the risk of wet AMD; **(F)**. The MR estimate for the effect of MN on the risk of wet AMD. CKD, chronic kidney disease; eGFRcrea, creatinine-based estimated glomerular filtration rate; eGFRcys, cystatinC-based estimated glomerular filtration rate; DN, diabetic nephropathy; immunoglobulin A nephropathy, IgAN; MN, membranous nephropathy; AMD, age-related macular degeneration; MR, Mendelian randomization; SNP, single nucleotide polymorphism.

Five MR methods provided no evidence of an association between CKD, eGFRcrea, eGFRcys, IgAN, and wet AMD (*p* > 0.05, Table 2). Except for the simple mode, the other four MR methods consistently showed that DN increases the risk of wet AMD (OR > 1, *p* < 0.05, Table 2). All five methods showed a causal relationship between MN and an increased risk of wet AMD (OR > 1, *p* < 0.05, Table 2). Figure 5 provided scatter plots showing the causal relationship between CKD and wet AMD. No significant heterogeneity and horizontal pleiotropy were found according to Cochrane’s Q, and MR-Egger (*p* > 0.05, Supplementary Table S2). The results of leave-one-out sensitivity and single SNP risk analysis were shown in Supplementary Figures S7, S8.

## 4 Discussion

In this study, we conducted a comprehensive MR analysis to investigate the causal impact of CKD on the risk of AMD. We did not find any association between CKD, eGFR, and any type of AMD (*p* > 0.05). However, DN and MN were associated with an increased risk of earl AMD. Furthermore, DN, IgAN, and MN were associated with an increased risk of AMD. Specifically, DN and IgAN were associated with an increased risk of dry AMD, while the risk of wet AMD was associated with DN and MN.

Several observational studies have shown that CKD increases the risk of AMD. For instance, Liew et al. conducted a population-based prospective cohort study involving 1,183 participants aged 54 and older. After adjusting for age, gender, smoking, and other risk factors, they found that individuals with moderate CKD (eGFR 30 to 59 mL/min per 1.73 m^2^) were three times more likely to develop early AMD than those with no or mild CKD (Liew et al., 2008). Similarly, Beaver Dam Eye Study showed that mild CKD (45–59 mL/min per 1.73 m^2^) was associated with early AMD, but not the incidence of exudative AMD and pure geographic atrophy or the progression of AMD (Klein et al., 2009). Furthermore, in another study involving 3,008 Koreans aged 50–87 found a significant association between CKD and early AMD (Choi et al., 2011). Notably, a case–control subset analysis of NHANES III demonstrated that a lower eGFR was independently associated with late AMD (Weiner et al., 2011). In a diverse population, a population-based cross-sectional study involving 9,799 participants from Chinese, Malay, and Indian ethnic groups in Singapore found that CKD was identified as a significant risk factor for late AMD, but not for early AMD (Cheung et al., 2014). Numerous other investigations also have found an association between CKD and AMD (Deva et al., 2011; Gao et al., 2011; Wang et al., 2016; Cheng et al., 2017; Leisy et al., 2017a,b). More recently, a comprehensive study that pooled data from 51,253 participants across 10 different Asian population-based studies found a significant association between CKD (and decreased kidney function) and late AMD. Contrastingly, the same study found no significant association between CKD and early AMD (Xue et al., 2023).

However, not all studies support an association between CKD and AMD. In a population-based cross-sectional study of individuals aged 75 and over in Britain, it was found that the association between reduced eGFR and AMD in men was not significant (Nitsch et al., 2009). Similarly, another cross-sectional study involving 5,874 participants (aged 45 to 84) indicated that there was no association between impaired kidney function and early AMD (Chong et al., 2014). Moreover, the Singapore Epidemiology of Eye Diseases (SEED, 2004–11) study revealed no link between CKD and AMD in adults aged 40–80 (Wong et al., 2016). In the same vein, Zhu *et al*’s investigation of 5,518 participants aged 40 or above in the NHANES 2005–2008 study found no correlation between CKD and AMD, even after accounting for multiple confounding factors (Zhu et al., 2020). And Dave *et al*’s research found no association between renal function parameters and AMD features, even after adjusting for age, as per their multimodal retinal imaging study (Dave et al., 2022).

In summary, whether a causal relationship exists between CKD and AMD remains uncertain. A significant reason for this uncertainty is that CKD and AMD share common risk factors, such as age, hypertension, obesity, smoking, and alcohol consumption (Wong C. W. et al., 2014). These factors concurrently promote the progression of both CKD and AMD, which could lead to the observed association between the two in epidemiological studies. The varying results of observational studies may also depend on the population studied, the definitions and rigor of AMD diagnosis and phenotypic assessments, as well as the sample sizes of the research groups. Moreover, it should be noted that in observational studies, CKD patients are defined as those with an eGFR <60 mL/min per 1.73 m^2^, and some studies stratify eGFR. However, these studies do not investigate the earlier stages of CKD, stages 1–2, and the disease spectrum of CKD varies among different populations. In Asian populations, chronic glomerulonephritis predominates, while in European populations, DN and hypertensive nephrosclerosis are more common. This could be another significant reason for the inconsistency in the results of observational studies that should not be overlooked.

In our study, we did not find an association between CKD, eGFRcys, eGFRcrea, and various types of AMD. CKD (defined as an eGFR below 60 mL/min/1.73 m^2^) is an umbrella term encompassing a variety of etiologies and pathological processes. Different CKD subtypes may have distinct risk factors and pathogenic mechanisms. In GWAS of CKD, treating CKD as a whole may obscure the associations between individual CKD subtypes and AMD, as the effects of these subtypes may not be significant within the overall CKD population. In GWAS of CKD, there is a lack of subgroup analysis data for specific causes such as DN, hypertensive kidney damage, obstructive nephropathy, kidney stones, and glomerulonephritis (MN, IgAN), which limits our further research. This absence of detailed analysis may be one of the reasons why our MR study did not find an association between CKD and AMD. MR analysis targeting common etiologies of CKD suggests that DN, IgAN, and MN can increase the risk of AMD. There have been no observational studies reporting on this aspect to date. Current research indicates that kidney diseases may promote the progression of AMD through multiple mechanisms. For instance, the critical role of the Renin-Angiotensin-Aldosterone System (RAAS) in the progression of renal damage in proteinuric kidney diseases has been extensively described. Upregulation of the RAAS can lead to endothelial dysfunction, inflammation, and oxidative stress (Izzedine et al., 2003). RAAS affects the blood flow in the retina, iris, and ciliary body, regulating intraocular pressure by altering the balance between aqueous humor production and outflow. The kidney and the eye share similarities in structure, development, and physiological pathways. Both organs possess extensive vascular networks. In fact, the inner retina and the glomerulus have similar filtration barriers that are regulated by the RAAS. Kidney diseases represented by DN, MN, and IgAN could promote the progression of ocular diseases such as AMD through the RAAS (Lai et al., 2022). Additionally, dysfunctions of the complement system (Zipfel et al., 2006), apolipoprotein E (Baird et al., 2004; Hsu et al., 2005) and atherosclerosis (Friedman, 2000) may also be mechanisms through which kidney diseases can promote the progression of AMD.

However, our study has certain limitations. Firstly, CKD is a relatively broad diagnosis. In clinical practice, it is still necessary to pay attention to the etiology and staging of CKD. The current GWAS data on CKD and eGFR lack information on the etiology and staging of CKD, which limits our further research. Secondly, our study is subject to a degree of sample overlap. The GWAS data for DN, wet AMD, and dry AMD are all from the FinnGen. Thirdly, it is imperative to exercise caution when extrapolating our findings to populations beyond the scope of this study, given that the GWAS datasets employed were limited to participants of European ancestry. Lastly, although we tried our best to exclude and correct potential confounders, there may still be other factors related to AMD that were not considered in the analysis. The IVs applied in our MR approach could still conceivably elevate the risk of AMD through pleiotropic effects that were not detected.

## 5 Conclusion

In conclusion, this MR study presents compelling evidence for a causal relationship between genetic predispositions to DN, MN, and IgAN, and a heightened risk of AMD. Conversely, the evidence is currently inadequate to confirm a causal connection between CKD, eGFR, and AMD. These results emphasize the significance of conducting ocular examinations in patients diagnosed with DN, MN, and IgAN. Additionally, our research underscores the need for further investigation into these associations using larger and more diverse cohorts.

## Data availability statement

The original contributions presented in the study are included in the article/Supplementary material, further inquiries can be directed to the corresponding author.

## Ethics statement

Ethical approval was not required for the study involving humans in accordance with the local legislation and institutional requirements. Written informed consent to participate in this study was not required from the participants or the participants’ legal guardians/next of kin in accordance with the national legislation and the institutional requirements.

## Author contributions

YH: Data curation, Methodology, Writing – original draft. QL: Data curation, Methodology, Visualization, Writing – review & editing. ZX: Methodology, Supervision, Visualization, Writing – review & editing. YL: Methodology, Supervision, Writing – review & editing. XT: Writing – review & editing. ZW: Methodology, Writing – review & editing.
