# Supplementary material for: Association between chronic kidney disease and age-related macular degeneration: a Mendelian randomization study
Source: Front Aging Neurosci. 2024 May 30;16:1399666. doi: 10.3389/fnagi.2024.1399666 (PMC11169943; doi:10.3389/fnagi.2024.1399666)
Supplement: Supplementary file 1 [file Data_Sheet_1.docx]

Supplementary Material

# Supplementary Figures and Tables

## Supplementary Tables

Supplementary Table S1. Summary of genome-wide association studies (GWAS) datasets in study.

| Trait | Year | Sample size | Population | Pubmed ID /Consortium |
| --- | --- | --- | --- | --- |
| CKD | 2019 | 480,698 | European | 31152163/ CKDGen |
| eGFRcrea | 2021 | 1,004,040 | European | 34272381/ CKDGen, UK Biobank |
| eGFRcys | 2021 | 460,826 | European | 34272381/ CKDGen, UK Biobank |
| DN | 2023 | 312,650 | European | FinnGen |
| IgAN | 2021 | 477,784 | European | 34594039/FinnGen, UK Biobank |
| MN | 2020 | 7,979 | European | 32231244 |
| early AMD | 2020 | 105,248 | European | 32843070/ IAMDGC, UK biobank |
| AMD | 2023 | 391,060 | European | FinnGen |
| dry AMD | 2023 | 279,155 | European | FinnGen |
| wet AMD | 2023 | 279,159 | European | FinnGen |

CKD, chronic kidney disease; eGFRcrea, creatinine-based estimated glomerular filtration rate; eGFRcys, cystatinC-based estimated glomerular filtration rate; DN, diabetic nephropathy; immunoglobulin A nephropathy, IgAN; MN, membranous nephropathy; AMD, age-related macular degeneration; IAMDGC, International AMD genomics consortium,

Supplement Table S2. The results of heterogeneity and horizontal pleiotropy analysis.

|  |  | Cochran’s Q test | | | MR-Egger intercept test | | |
| --- | --- | --- | --- | --- | --- | --- | --- |
| Exposure | Outcome | Q | df | *P* value | Egger intercept | SE | *P* value |
| CKD | early AMD | 14.079 | 16 | 0.593 | 0.015 | 0.017 | 0.369 |
| eGFRcrea | early AMD | 230.174 | 295 | 0.998 | -0.003 | 0.002 | 0.143 |
| eGFRcys | early AMD | 116.245 | 162 | 0.997 | 0.002 | 0.002 | 0.361 |
| DN | early AMD | 5.816 | 8 | 0.668 | 0.005 | 0.014 | 0.715 |
| IgAN | early AMD | 6.057 | 7 | 0.533 | -0.011 | 0.025 | 0.685 |
| MN | early AMD | 12.253 | 14 | 0.586 | -0.017 | 0.012 | 0.162 |
| CKD | AMD | 15.067 | 14 | 0.374 | 0.001 | 0.002 | 0.762 |
| eGFRcrea | AMD | 272.273 | 277 | 0.569 | -0.005 | 0.003 | 0.087 |
| eGFRcys | AMD | 131.924 | 146 | 0.792 | 0.040 | 0.018 | **0.045** |
| DN | AMD | 5.087 | 8 | 0.748 | -0.021 | 0.015 | 0.197 |
| IgAN | AMD | 2.731 | 4 | 0.604 | 0.027 | 0.026 | 0.379 |
| MN | AMD | 8.576 | 10 | 0.573 | -0.027 | 0.016 | 0.117 |
| CKD | dry AMD | 12.205 | 15 | 0.663 | -4.50E-04 | 0.003 | 0.879 |
| eGFRcrea | dry AMD | 252.628 | 277 | 0.851 | -0.006 | 0.003 | 0.074 |
| eGFRcys | dry AMD | 117.268 | 145 | 0.956 | 0.034 | 0.021 | 0.139 |
| DN | dry AMD | 3.256 | 8 | 0.917 | -0.015 | 0.018 | 0.427 |
| IgAN | dry AMD | 1.417 | 4 | 0.841 | 0.013 | 0.034 | 0.716 |
| MN | dry AMD | 14.363 | 13 | 0.349 | 0.003 | 0.017 | 0.880 |
| CKD | wet AMD | 10.558 | 15 | 0.783 | 0.017 | 0.029 | 0.559 |
| eGFRcrea | wet AMD | 232.487 | 278 | 0.978 | 0.001 | 0.003 | 0.854 |
| eGFRcys | wet AMD | 126.603 | 153 | 0.941 | -0.007 | 0.003 | 0.053 |
| DN | wet AMD | 11.239 | 8 | 0.189 | -0.03 | 0.023 | 0.226 |
| IgAN | wet AMD | 4.111 | 5 | 0.534 | -0.004 | 0.035 | 0.909 |
| MN | wet AMD | 5.852 | 11 | 0.883 | -0.032 | 0.020 | 0.129 |

CKD, chronic kidney disease; eGFRcrea, creatinine-based estimated glomerular filtration rate; eGFRcys, cystatinC-based estimated glomerular filtration rate; DN, diabetic nephropathy; IgAN, immunoglobulin A nephropathy; MN, membranous nephropathy; AMD, age-related macular degeneration.

## 1.2 Supplementary Figures


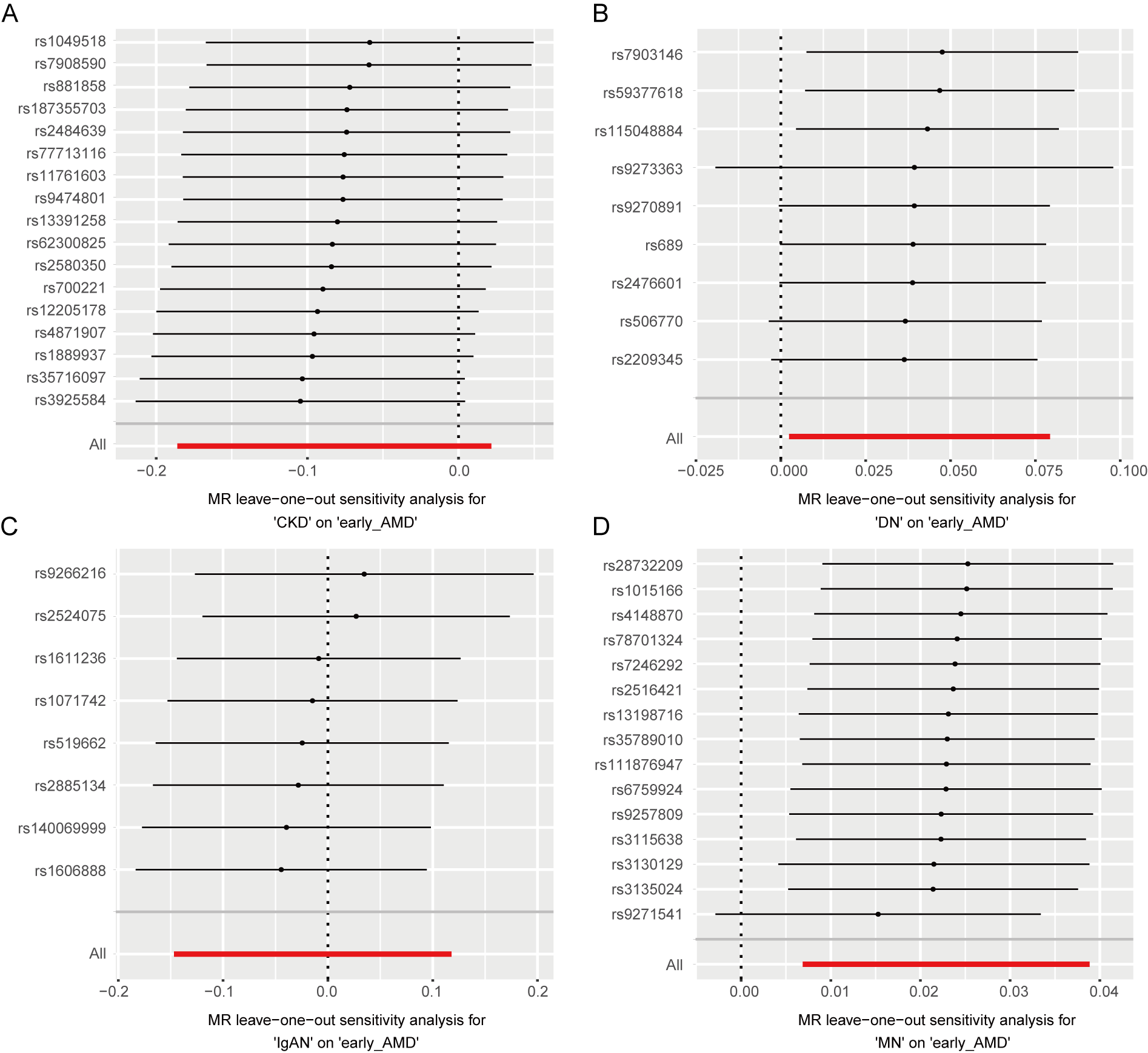


Supplementary Figure S1. Leave-one-out plots of CKD with the risk of early AMD. A. Leave-one-out analysis of the causal association between CKD and early AMD; B. Leave-one-out analysis of the causal association between DN and early AMD; C. Leave-one-out analysis of the causal association between IgAN and early AMD; D. Leave-one-out analysis of the causal association between MN and early AMD. CKD, chronic kidney disease; DN, diabetic nephropathy; IgAN, immunoglobulin A nephropathy; MN, membranous nephropathy; AMD, age-related macular degeneration.


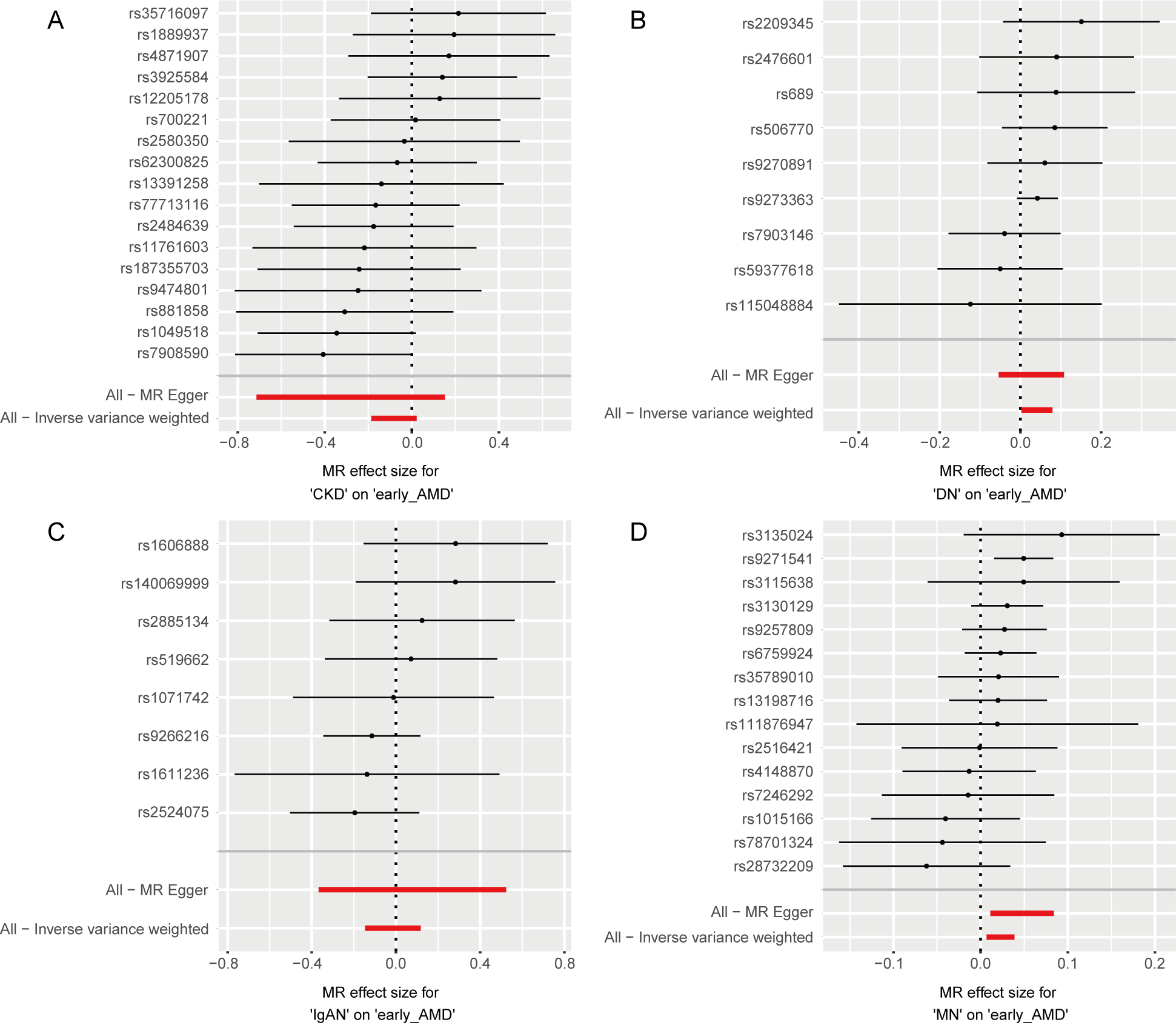


Supplementary Figure S2. The forest plot of the causal effect of CKD on early AMD. The effect of each SNP was calculated separately, and the overall effect was calculated using MR Egger and IVW methods. A. The forest plot of the causal effect of CKD on early AMD; B. The forest plot of the causal effect of DN on early AMD; C. The forest plot of the causal effect of IgAN on early AMD; D. The forest plot of the causal effect of MN on early AMD. CKD, chronic kidney disease; DN, diabetic nephropathy; IgAN, immunoglobulin A nephropathy; MN, membranous nephropathy; AMD, age-related macular degeneration.


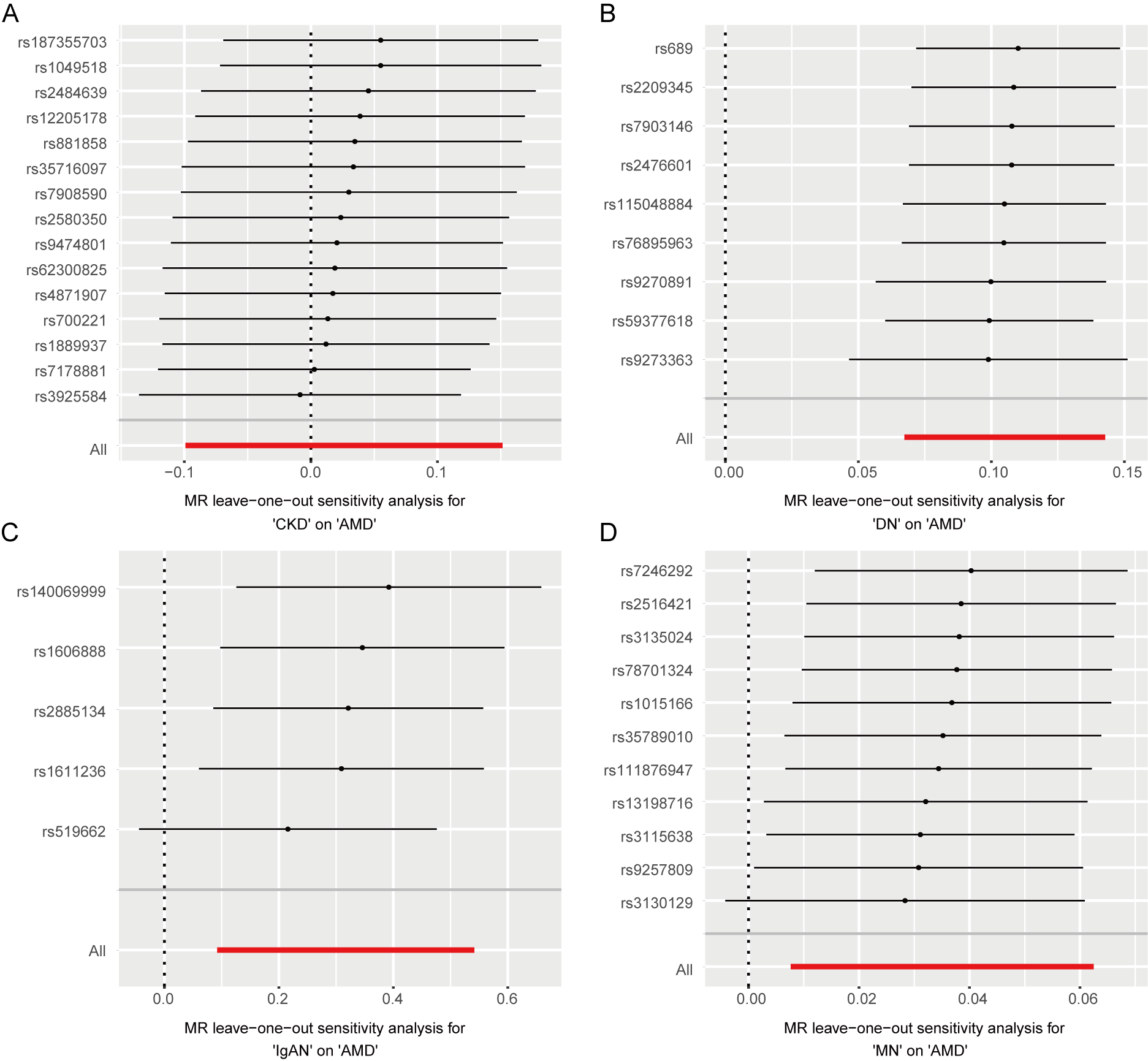


Supplementary Figure S3. Leave-one-out plots of CKD with the risk of AMD. A. Leave-one-out analysis of the causal association between CKD and AMD; B. Leave-one-out analysis of the causal association between DN and AMD; C. Leave-one-out analysis of the causal association between IgAN and AMD; D. Leave-one-out analysis of the causal association between MN and AMD. CKD, chronic kidney disease; DN, diabetic nephropathy; IgAN, immunoglobulin A nephropathy; MN, membranous nephropathy; AMD, age-related macular degeneration.


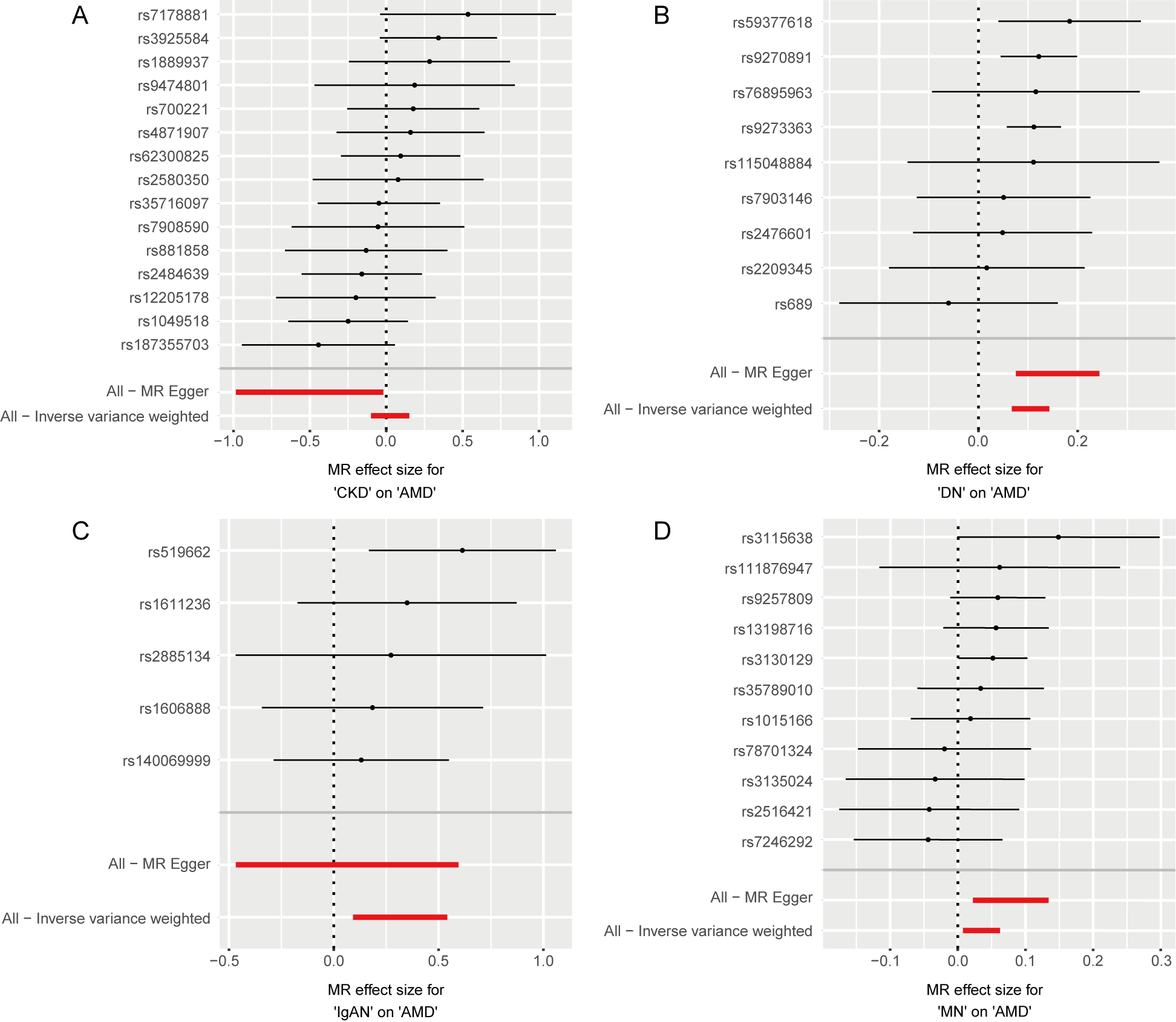


Supplementary Figure S4. The forest plot of the causal effect of CKD on AMD. The effect of each SNP was calculated separately, and the overall effect was calculated using MR Egger and IVW methods. A. The forest plot of the causal effect of CKD on AMD; B. The forest plot of the causal effect of DN on AMD; C. The forest plot of the causal effect of IgAN on AMD; D. The forest plot of the causal effect of MN on AMD. CKD, chronic kidney disease; DN, diabetic nephropathy; IgAN, immunoglobulin A nephropathy; MN, membranous nephropathy; AMD, age-related macular degeneration.


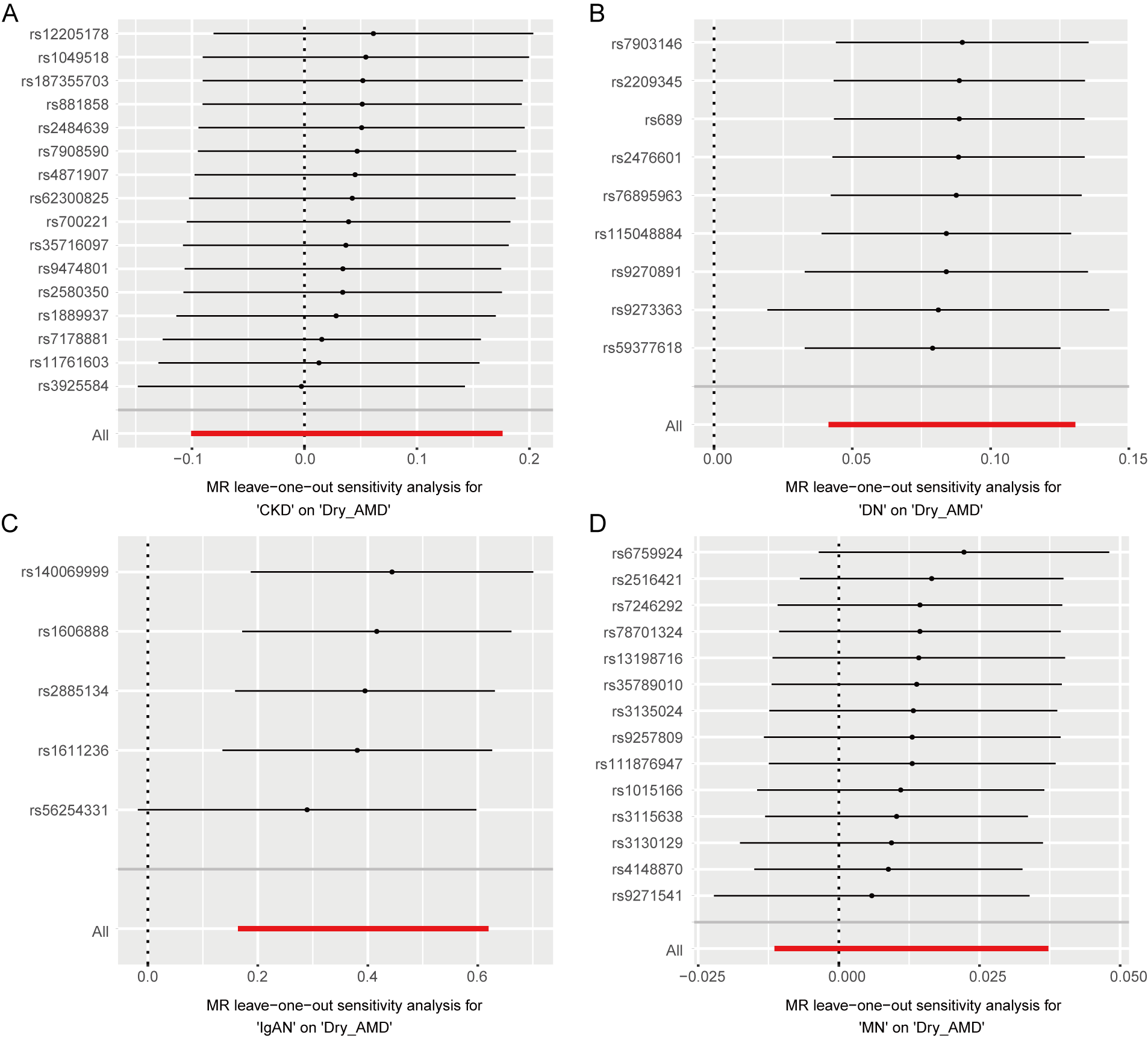


Supplementary Figure S5. Leave-one-out plots of CKD with the risk of dry AMD. A. Leave-one-out analysis of the causal association between CKD and dry AMD; B. Leave-one-out analysis of the causal association between DN and dry AMD; C. Leave-one-out analysis of the causal association between IgAN and dry AMD; D. Leave-one-out analysis of the causal association between MN and dry AMD. CKD, chronic kidney disease; DN, diabetic nephropathy; IgAN, immunoglobulin A nephropathy; MN, membranous nephropathy; AMD, age-related macular degeneration.


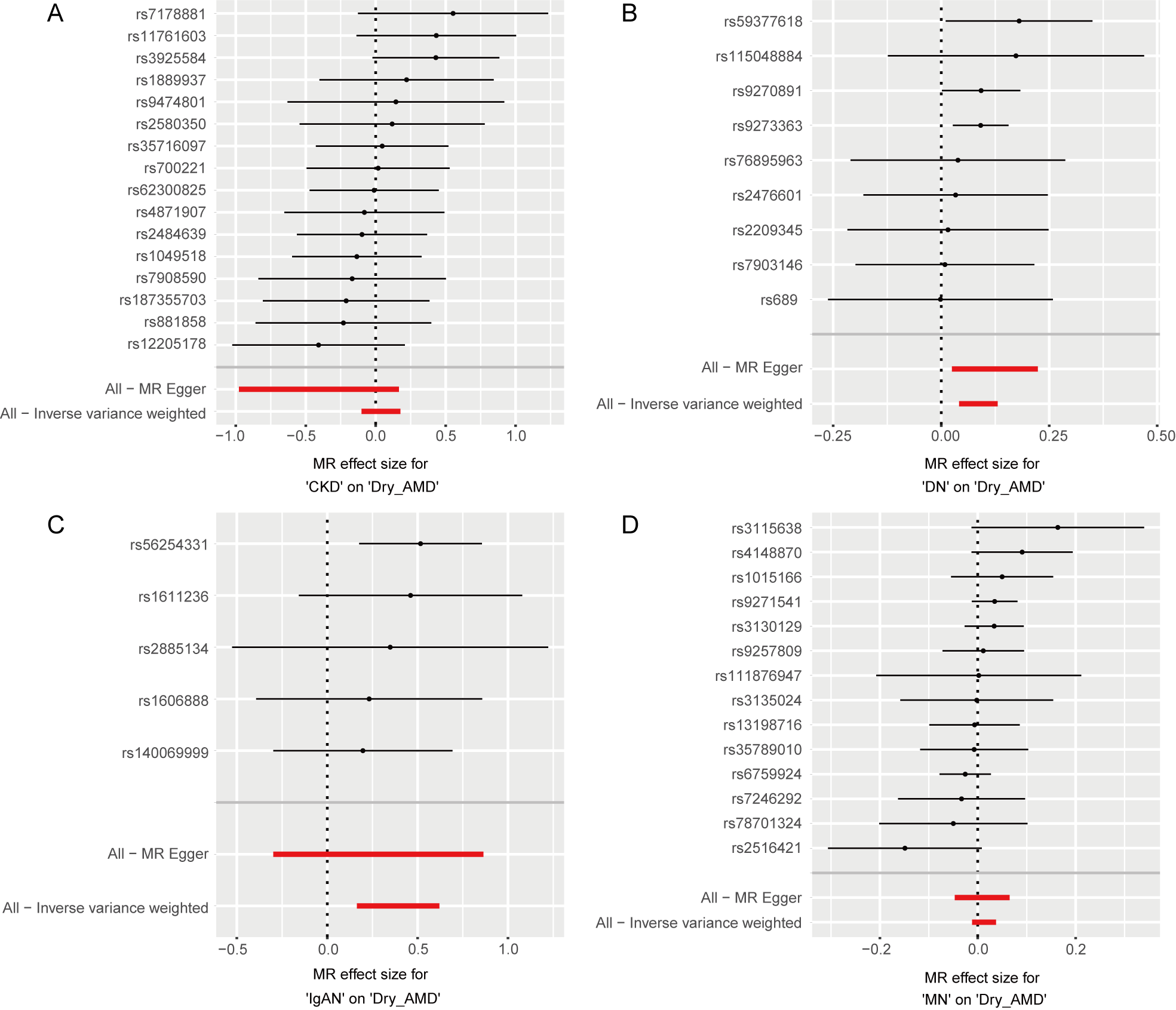


Supplementary Figure S6. The forest plot of the causal effect of CKD on dry AMD. The effect of each SNP was calculated separately, and the overall effect was calculated using MR Egger and IVW methods. A. The forest plot of the causal effect of CKD on dry AMD; B. The forest plot of the causal effect of DN on dry AMD; C. The forest plot of the causal effect of IgAN on dry AMD; D. The forest plot of the causal effect of MN on dry AMD. CKD, chronic kidney disease; DN, diabetic nephropathy; IgAN, immunoglobulin A nephropathy; MN, membranous nephropathy; AMD, age-related macular degeneration.


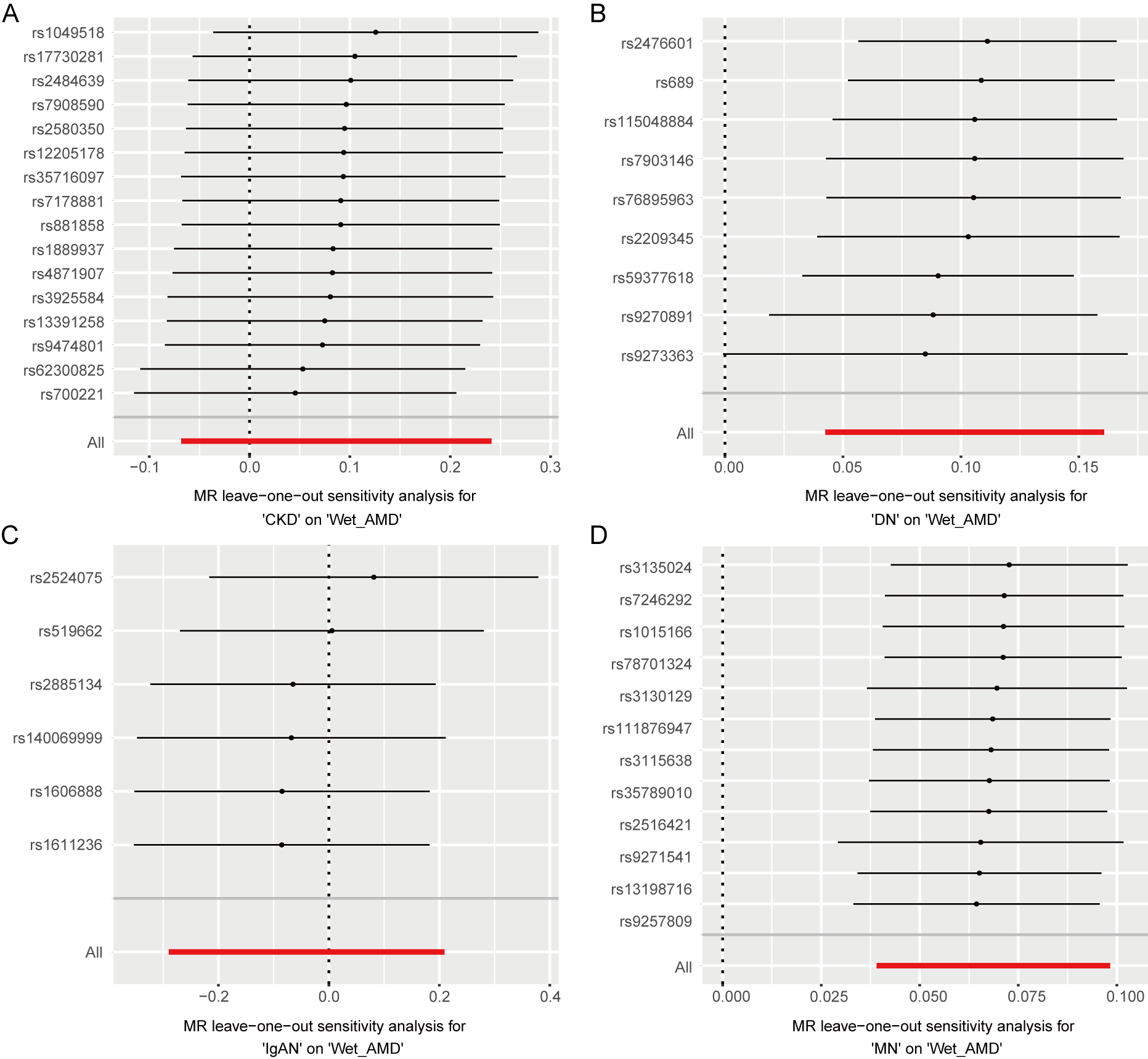


Supplementary Figure S7. Leave-one-out plots of CKD with the risk of wet AMD. A. Leave-one-out analysis of the causal association between CKD and wet AMD; B. Leave-one-out analysis of the causal association between DN and wet AMD; C. Leave-one-out analysis of the causal association between IgAN and wet AMD; D. Leave-one-out analysis of the causal association between MN and wet AMD. CKD, chronic kidney disease; DN, diabetic nephropathy; IgAN, immunoglobulin A nephropathy; MN, membranous nephropathy; AMD, age-related macular degeneration.


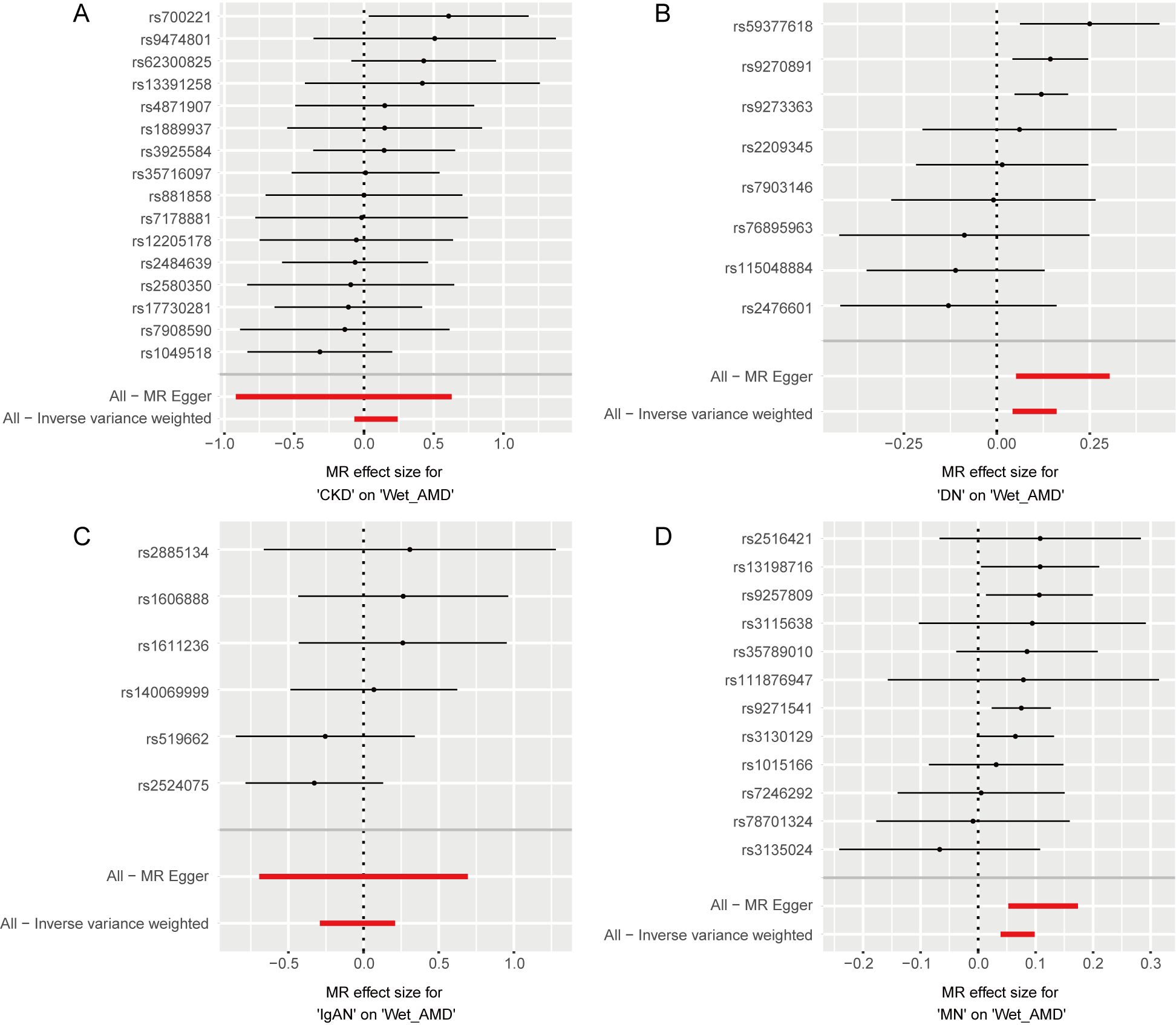


Supplementary Figure S8. The forest plot of the causal effect of CKD on wet AMD. The effect of each SNP was calculated separately, and the overall effect was calculated using MR Egger and IVW methods. A. The forest plot of the causal effect of CKD on wet AMD; B. The forest plot of the causal effect of DN on wet AMD; C. The forest plot of the causal effect of IgAN on wet AMD; D. The forest plot of the causal effect of MN on wet AMD. CKD, chronic kidney disease; DN, diabetic nephropathy; IgAN, immunoglobulin A nephropathy; MN, membranous nephropathy; AMD, age-related macular degeneration.
